# Evaluation on interactive waiting experience design of mobile internet products based on machine learning

**DOI:** 10.1038/s41598-023-43405-2

**Published:** 2023-10-09

**Authors:** Yi Yu

**Affiliations:** Artistics, Krirk University, Bangkhen, Bangkok, 10220 Thailand

**Keywords:** Biological techniques, Systems biology

## Abstract

In today’s rapidly changing economy, efficient lifestyle has become the current situation of most mobile product users. With the development of performance tools and technologies, a fast lifestyle has brought more wealth and opportunities to users. The slow pace and fluctuating time are indirect income losses, which cause user anxiety to some extent. When the waiting time exceeds the user’s waiting threshold, users would experience negative emotions, such as boredom, anxiety and anger, and product satisfaction would drop significantly. Therefore, by analyzing the uniqueness of mobile Internet products and the characteristics of users, this paper studied the reasons and influencing factors of product interactive waiting, and then used machine learning algorithm to analyze the cost function of interactive waiting experience. Finally, the corresponding interactive waiting experience design strategy was proposed. By comparison, the user experience after product interaction optimization design was 8.4% higher than that before product interaction optimization design, and the user frequency was also 14.7% higher after optimization design. In short, user experience plays an important role in product interaction design.

## Introduction

With the rapid development of smart devices and mobile applications, the portability and miniaturization of mobile Internet products have been supported by most users, creating a huge space for mobile application development. Due to the uneven configuration of mobile devices and a large number of information processing algorithms, waiting for information on the page is a major challenge for users. At the same time, in the context of rapid improvement of information processing technology and hardware performance, users have increasingly high requirements for interaction with applications. Therefore, the design of interactive products not only focuses on the structure and organization of the elements of the software itself, but also focuses on the changes of users’ behavior and emotion in the process of use.

User experience plays an important guiding role in product design. Tao Fei proposed a new method of product design based on the digital twin method, introduced the development of product design, and then proposed and analyzed the framework of digital twin-driven product design^1^. Sousa Rui studied the relationship between product customization and service strategy, especially the relationship between the strength of product customization strategy and the degree of service, and the regulatory effect of product customization strategy consistency on this relationship^2^. Petit Olivia believed that sensory marketing in the digital environment can provide a richer online experience, which was expected to open the way for further research and development in this field^3^. Hoyer Wayne D provided a new type of AI driven new technology to manage these new AI technologies along the customer experience dimension to create experience value^4^. Bleier Alexander examined the role of unique design elements in shaping the four dimensions of the online customer experience. Product experience and brand reputation characteristics exacerbate or reduce the inherent uncertainty of online shopping, thereby reducing the impact of various experience dimensions on purchases^5^. Izogo Ernest Emeka provided a comprehensive and dynamic customer experience model, covering all stages of the customer decision-making process. By drawing on new theoretical perspectives, he demonstrated the unique properties of the online shopping experience that contributed to existing research on customer experience^6^. Chen Ja-Shen proposed a research model based on technology acceptance model and information system success model to describe the relationship among chat robot adoption, online customer experience and customer satisfaction^7^. The above studies have described the importance of user experience, but there are still some deficiencies in interactive waiting experience.

Many scholars have studied machine learning and analyzed the quality of user experience. Nwakanma Cosmas Ifeanyi outlined the potential of using natural language processing systems to classify user experience quality, and developed an improved regression classifier to test, train and classify user experience^8^. Yi Shanshan applied machine learning algorithm to analyze product information and store information based on customer experience learning, and collected product data with customer comments from the benchmark unified computing system^9^. Chi-Hsien Kuo built a machine learning model to help minimize research barriers, and proposed an unsupervised end-to-end model that can directly and accurately handle user experience effects without human intervention^10^. Liu Lina used ontology to build a user knowledge model, integrated multiple situational similarity indicators, and studied the artistic design of user interaction experience in mobile systems through situational awareness and machine learning methods^11^. The above studies have described the user experience effect, but not the experience design strategy.

In order to study the interactive waiting experience of mobile internet products, this paper analyzed and studied the cost function of interactive waiting experience through machine learning, and then analyzed the factors of interactive waiting experience. Finally, the strategy was proposed and experimental analysis was carried out. According to the experimental analysis, users were satisfied with the optimization of interactive waiting experience design, and the waiting time and user experience have been greatly improved. Compared with other literature, this paper focused on the use of experimental analysis of user waiting time and experience before and after optimization.

## Mobile internet product uniqueness and user evaluation

### Uniqueness of mobile internet products

The uniqueness of mobile internet products can be analyzed from two aspects. The first is the difference between mobile internet and website. Mobile Internet has the characteristics of mobility, real-time, accuracy and positioning. Compared with network products with fixed network location and fixed network as the main scenarios, the use scenarios of mobile internet products are very diverse. The transmission environment and size of mobile internet products are limited to networks and terminals, processing capacity and battery capacity. Therefore, the difference between mobile internet and web pages is that mobile internet takes mobile scenario as its main use scenario. Users can use any operation at anytime and anywhere, and the functions of mobile products are always easy to distribute and centralize information. Customers are segmented according to participation and use of discretion while paying attention to the satisfaction level of different market segments^12^. Secondly, the waiting status of mobile internet products is different from that of network products. Compared with Internet products, the interactive waiting state of the mobile Internet has the following characteristics, but users’ tolerance for the waiting state of mobile products is lower than that of Internet products, making the waiting state of mobile products mainly short-term downloads. Compared with various operation modes that need to be executed on the website (such as downloading and a large amount of information input), mobile internet products use output type as the main operation mode and read related operations as the main behavior, so most of the download operations occur on page separators and content downloads.

### User evaluation of mobile internet products

**Figure 1:**
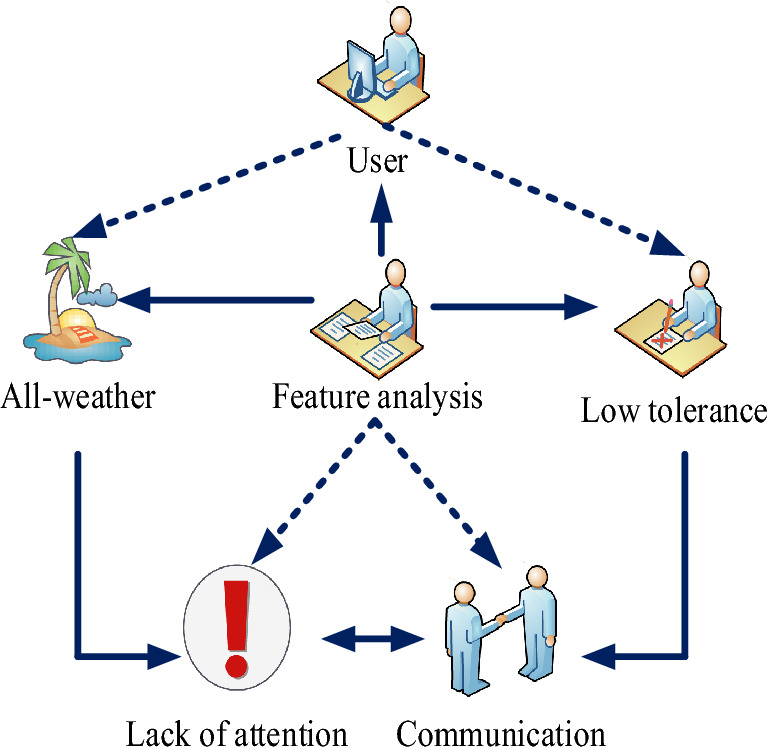
User analysis of mobile internet products.

According to the analysis of users using mobile internet products, these users have common characteristics, as shown in Fig. 1. The first is all-weather. Mobile Internet exists in all areas of people’s life. Mobile Internet users work 24 h a day, from morning to night. Mobile Internet has become the choice of most users. They can easily use mobile phones or tablets to avoid external interference. The second is low tolerance. Mobile products have many users and different types of applications. Due to the complex scene or low tolerance of mobile devices, users use mobile products with less user experience. Due to the small problem of the application, enterprise users have abandoned their products and can use other products, which also puts forward high requirements for the user experience design of mobile Internet products. The third is the lack of attention. Mobile products are small and easy to carry and use. Therefore, if users use the product for other purposes or mobile Internet products and use the mobile Internet for free again, other things may happen suddenly. If they are not concentrated, they are prone to misoperation. The fourth is communication and exchange. With the widespread use of mobile internet, social media applications are not enough. AI can affect the way products interact with customers^13^. Social software can chat with friends at anytime and anywhere. The most important thing is to spread information. A good user experience can increase the number of users.

## Status evaluation of interactive waiting experience of mobile internet products

### Reasons for interactive waiting status of mobile internet products

**Figure 2:**
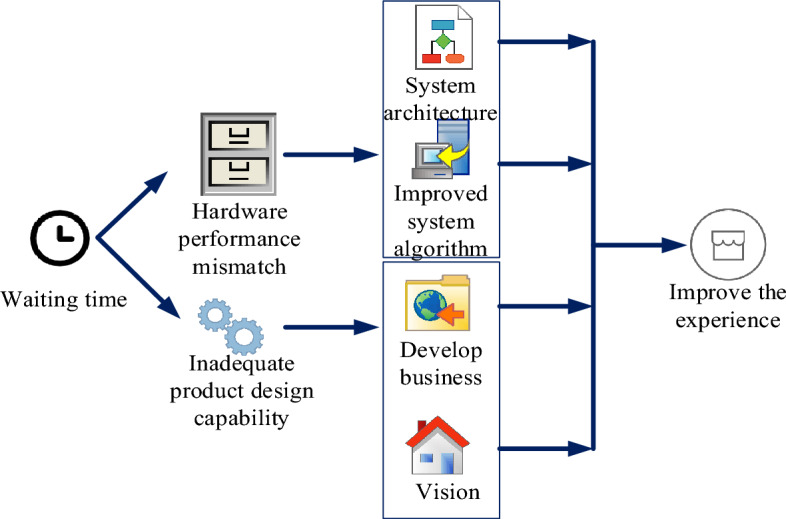
Reasons for interactive waiting status of mobile internet products.

Although mobile Internet hardware and related technologies continue to develop rapidly, every technological progress is determined by users, and the huge development of technical capabilities is always accompanied by huge demand. When the requirements are not met, the technology use process would occur, resulting in the “waiting” phenomenon. The reasons can be summarized as two points, as shown in Fig. 2.

First, the performance of software and hardware should not match the interactive waiting state caused by data requirements. Secondly, the mobile product design capacity is insufficient. In the first case, people can not only improve this situation by investing in software and hardware, but also fundamentally solve the problem by improving the system algorithm and the system architecture of development information and user cognitive verification. People can design interventions to improve the user’s waiting experience. For the latter, business, process, logic, behavior, vision and other elements waiting for interactive design can be developed to optimize the whole work.

As for the appearance of interactive waiting state, due to the difference of system performance, its existence cannot be recognized. In addition, interactive waiting should not be regarded as a waste of time and a reduction of product user experience. The designer pays attention to the humanized user experience perspective, optimizes the detailed waiting chain, alleviates users’ concerns through online applications and reviews the online waiting status. The standby state must be friendly designed to adapt to the user environment. Frequently, long wait and other unplanned wait can affect the design scenario, which needs to be optimized and improved. The objective performance of hardware and software directly determines the work efficiency of users when using mobile products. Better hardware quality and optimized software algorithms can effectively shorten the waiting time of users when using products and improve the use efficiency.

### Factors affecting interactive waiting of mobile internet products

**Figure 3:**
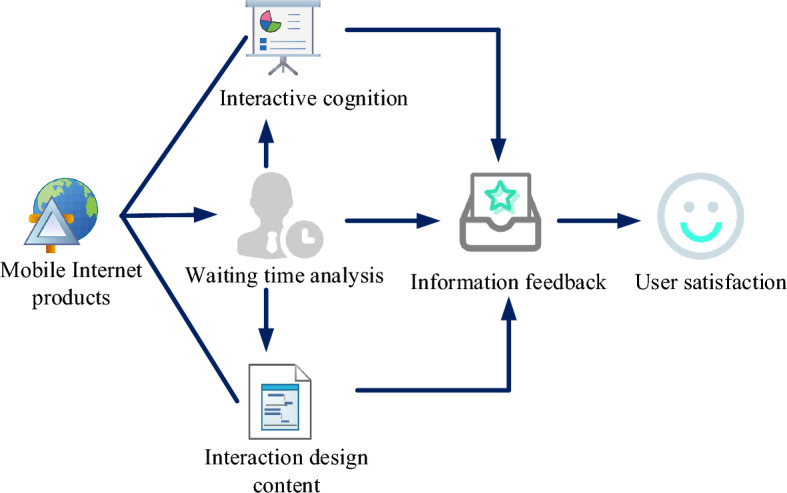
Factors affecting interactive waiting of mobile internet products.

The factors affecting the interactive waiting of mobile Internet products are mainly divided into two categories, as shown in Fig. 3.

The first is the cognitive node of the interaction process. Once users are stimulated by information on the interface, they would develop knowledge about the interface, and the interface would understand feedback and cooperate with them. The expectation of mobile products depends on all these cognitive processes. If it is difficult to read the interface information at the sensitive stage of the interface content, the efficiency of users accessing information through the interface would be greatly reduced, which would affect the interaction efficiency. The user’s understanding of information is mobile, which directly affects the interaction efficiency of the product and the understanding and readability of the content. Users can quickly log in and run links, improve the interaction efficiency, reasonably apply interdisciplinary knowledge and technology, optimize the interaction waiting process, and make users feel humanized when using the system. Effective feedback has a great impact on the next interaction process. It hinders the interaction between users and the expected psychology of interaction, and increases the proportion of expected information in the interaction feedback.

The second is the content of interaction design. If there is no reasonable and humanized practical planning, it would lead to users’ perception of product information and related logic, which can affect the efficiency before user interaction, and thus affect the entire interaction process. This would lead to the passivation and maintenance of the map in the process of user interaction. The process of high-level data translation would directly affect the efficiency of user interaction. Due to the imperfect design, users inevitably show a “slow” and “stuck” cognitive waiting state before interaction, which would have a negative impact on the entire interaction process.

### Impact evaluation of interactive waiting experience of mobile internet products

**Figure 4:**
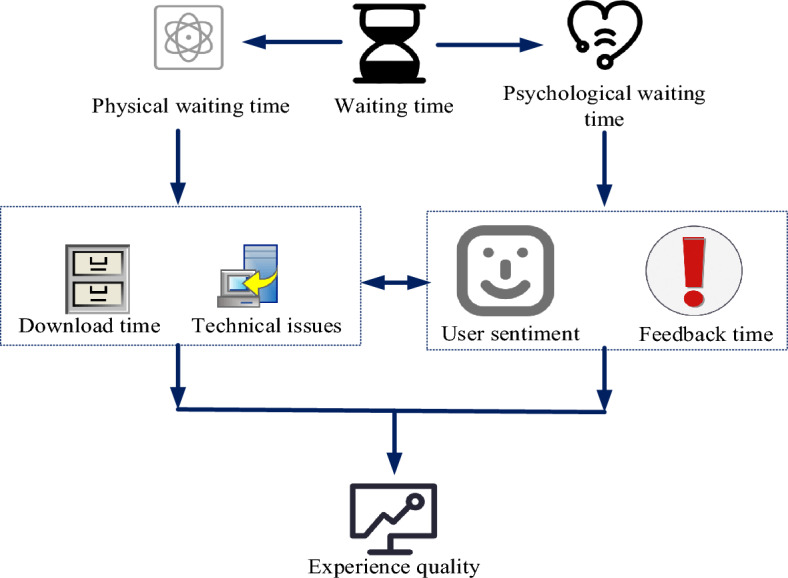
Impact analysis of interactive waiting experience of mobile internet products.

There are two types of interactive waiting time: physical waiting time and psychological waiting time, as shown in Fig. 4. Physical waiting time is the time information that can be measured using a time tool called target waiting time. It is objective and not affected by external factors. The psychological waiting time depends on the user’s subjective perception of time information, which depends on many external factors.

#### Objective factors

The objective factor is mainly the factor that the application product takes time to download, such as the network environment where the mobile device is located, mobile equipment installation, etc. All these processes would affect the software download time. Due to the uncertainty and technical limitations associated with the use of mobile products, it is often difficult to eliminate delays. For example, the background architecture method and other methods objectively affect the waiting time of users.

#### Subjective factors

The subjective factor is mainly the personal factor that affects the perception of download time when using the product. The user’s perception of waiting time caused by the subjective factor is usually called waiting time perception, that is to say, compared with the actual waiting time, it is the duration of the user’s subjective psychological perception. The understanding of waiting time is generally slightly different from the actual waiting time. On the one hand, these differences are affected by subjective factors. On the other hand, this in turn would have a negative impact on users, which would have some emotional impact on users. For people with high desire for control, products have a stronger positive impact on user experience^14^. Negative emotions would make users feel that the waiting time is longer than the actual time, thus further intensifying the negative emotions of users.

Common factors that affect users’ awareness of waiting time include emotion prolongs users’ awareness of waiting time. The user is busy performing urgent tasks, and the application feedback time is short, which affects the user’s understanding of the actual waiting time. Users who are familiar with the application may have relatively accurate psychological expectations, while users who are not familiar with the application may have higher expectations. These different psychological expectations directly affect their perception of application feedback time. When users wait for a long time, users would have negative emotions, which would affect their satisfaction with the product. These negative emotions would affect their perception of expectations. The longer the waiting time, the lower the user satisfaction with the product and the worse the user environment. The shorter the detection waiting time, the higher the user satisfaction with the product, and the better the user experience. If the perceived waiting time of users is less than the actual waiting time, their satisfaction with mobile products would be significantly improved.

## Application of machine learning lgorithm in intaeractive waiting experience of mobile internet products

Machine learning is a multidisciplinary discipline, involving probability theory, statistics, approximation theory, convex analysis, algorithm complexity theory and many other disciplines. It specializes in the study of how computers simulate or implement human learning behaviors to acquire new knowledge or skills, reorganize existing knowledge structures, and continuously improve their own performance. Machine learning algorithms can be classified according to different criteria. For example, according to the function f (x, θ), machine learning algorithms can be divided into linear models and nonlinear models. The machine learning algorithm used in this paper analyzes the cost function of interactive waiting experience.

In order to study the design effect of interactive waiting experience of mobile internet products, this paper analyzes the asymmetric probability and Euclidean distance in interactive waiting experience through machine learning algorithm, and then obtains the waiting probability and cost function of product interactive waiting experience design^15^. First, this paper calculates the asymmetric probability in the interactive waiting experience as follows:

1 $$a_{ij} = \frac{{\exp \left( { - s_{ij}^{2} } \right)}}{{\sum\nolimits_{k \ne i} {\exp \left( { - s_{ij}^{2} } \right)} }}$$

2 $$s_{ij}^{2} = \frac{{y_{i} - y_{j}^{2} }}{{2\alpha_{i}^{2} }}$$

Among them, $s_{ij}$ is the European distance in the interactive waiting experience; $y_{i} ,y_{j}$ are the maximum waiting time and the minimum waiting time, and $\alpha_{i}$ is the Gaussian kernel of the interactive waiting experience. Next, the waiting probability of interactive experience design is calculated using Gaussian neighborhood:

3 $$b_{ij} = \frac{{\exp \left( { - c_{i} - c_{j}^{2} } \right)}}{{\sum\nolimits_{k \ne i} {\exp \left( { - c_{i} - c_{k}^{2} } \right)} }}$$

Among them, *k* is the constant set manually, and $c_{i} ,c_{j}$ are the similar waiting description value in the Gaussian neighborhood. The final cost function of interactive waiting experience is:

4 $$T = \sum\limits_{i} {\sum\limits_{j} {a_{ij} \log \frac{{a_{ij} }}{{b_{ij} }}} }$$

The cost cost function is to measure the specific application effect of mobile Internet in product interactive waiting experience design, and evaluate the product interactive waiting experience design according to the change of cost function value before and after the use of mobile Internet.

## Design strategy for interactive waiting experience of mobile internet products

Through the interactive waiting cost function analyzed by machine learning algorithm, this paper proposed the following design strategies to optimize the interactive waiting experience of products. Based on the analysis of the influencing factors of interactive waiting experience, machine learning is used to analyze the cost function of interactive waiting experience, and then optimized design is carried out to improve the interactive waiting experience and shorten the waiting time of users.

### Optimizing loading feedback

When the product loading page, the user cannot detect the background operation and data loading process of the product^16^. If the user encounters any problem without interface feedback, the waiting process is monotonous, and the loading state and loading time are unknown, which directly leads to the user’s illusion of time estimation and prolongs the perception of waiting time. It returns to the page in an empty standby state, and displays the working status and the exact time when the user page loads in the background in the form of text or pictures, reflecting the current loading status and loading level. The waiting time can be determined in advance, thus reducing the change of user mood to an unknown state. Effective provision also facilitates the report on page loading status and the multi-channel communication model for downloading information to a certain extent.

### Alleviating user anxiety

During the user waiting period, in addition to the user’s immediate feedback on the product function, other negative emotions would be encountered, such as anxiety, fatigue and others. In order to meet the needs of users, designers often upload some content to help users browse the next page, so as to reduce the negative emotions caused by waiting and reduce users’ perception of time. In addition, the download speed is accelerated by reducing the quality of images or videos, and the data is downloaded gradually during viewing to ensure a smooth user experience.

### Providing alternative solutions for interactive waiting

If the user’s network environment is poor or the hardware configuration is low, the waiting time for application interaction is far longer than normal interaction, but these objective factors would not reduce the user experience or hinder normal use. It is recommended to further optimize the current online waiting. It is necessary to delete images and videos downloaded for a long time, or change the appearance of simple applications to reduce the quality of downloaded images^17^. In addition, there is a simple version of the system, which can directly develop the entire software set. In other words, application developers can remove complex and rare functions and only retain the basic functions that users usually use. By observing the concept of humanized design, and weighing advantages and disadvantages, they can make appropriate decisions to effectively improve the user experience.

### Compensation for excessive interaction waiting time

If the interactive waiting time is much longer than the user’s expectation, the user would have the idea of giving up waiting. Their efforts would not get a quick response, and their hearts would inevitably be unbalanced, which can measure whether it is necessary to wait for rewards. Once experiencing the product, all users can remember to start and end the experience. The proportion of good and bad experiences and the duration of the experience have a relatively small impact on the user’s memory. Therefore, compensating interactive waiting time can create human experience at the peak time of user waiting period, compensate user’s psychological bias, and reduce the impact of excessive waiting on user’s mood.

### Focusing on user needs

The user waiting time needs to be reduced. The application interface shows users how to predict the waiting time in advance and improve user satisfaction through interactive waiting. Interactive waiting should also focus on the concept of user experience design, and interactive waiting design must follow the user-oriented experience. From the perspective of system self-awareness and user interaction, it conforms to the user’s usage habits, expected interaction mode and corresponding psychological experience in a larger range. The effect of customer participation is created by enhancing gender voice and product intelligence^18^. In order to be truly user-centric, designers must put themselves in the scene where users are waiting, and experience users’ feelings and perceptions through observation, understanding, expected behavior and operating habits. Then the corresponding interactive waiting design is carried out to provide users with a more convenient experience.

The first step is to understand the user community. The user group here includes different users’ age, educational background, occupation, region and other factors. By understanding user groups, product design and interaction can be optimized according to their needs, habits, cognitive abilities, etc. Secondly, in the process of product interaction design, it should pay attention to concise and clear operation steps. An excellent Internet product should avoid lengthy and complex operations as much as possible, so that users can quickly and simply achieve the desired results when using it. At the same time, users should also be able to clearly understand the process and results of each step when interacting, so as to improve the operability of the product. Thirdly, product design should pay attention to record the user’s history of use records and behavior habits. This is conducive to establishing a more intimate connection with the user, and it can also be personalized and recommended in the subsequent design. Finally, a good Internet product should be differentiated as much as possible to highlight the features, so that users are easier to distinguish and remember. In all kinds of imitation and homogenization of the market, only products with characteristics can attract more users, so as to enhance the market competitiveness of products. For example, when King Glory matches players waiting to enter the game, Tencent gave some game guides in the interface to attract users’ attention and reduced the user’s feeling of waiting.

## Experimental evaluation of interactive waiting experience design of mobile internet products

In order to study the design effect of interactive waiting experience of mobile internet products, this paper analyzed the cost function of interactive waiting experience through machine learning, then compared the proportion of waiting time and information feedback effect before and after optimization, and finally compared the user experience and user use frequency before and after optimization. To this end, this paper first investigated the satisfaction of users in three regions after the interactive waiting design, including 50 people in each region. In this experiment, the traditional product interaction waiting experience is before optimization, and the optimized product interaction waiting experience design is under mobile Internet. The specific results are shown in Table 1.

**Table 1:** Satisfaction of users in three regions after interaction waiting design.

	Satisfied	Commonly	Dissatisfied	P-value
Region 1	37	8	5	0.025
Region 2	40	6	4	0.04
Region 3	41	7	2	0.035
Total	118	21	11	

By comparing the difference between the sample observations and the hypothesized population parameters, a statistic is calculated, and based on that statistic, a p-value is calculated to evaluate how credible the observations are under the hypothesis. If the p-value is less than a pre-set significance level (usually 0.05), the hypothesis is rejected as a significant difference in observations. It can be clearly seen from Table 1 that the P-value is less than 0.05, indicating that there is a dominant difference in the observed results.

**Figure 5:**
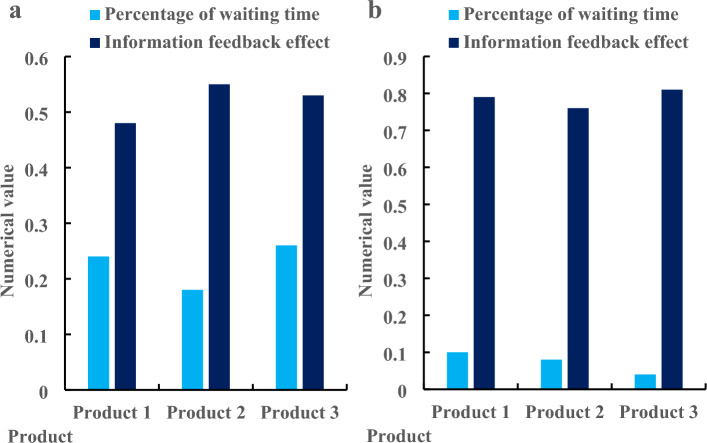
Proportion of waiting time and information feedback effect of users before and after interactive design of mobile internet products. (**a**) Before product interaction design optimization. (**b**) After product interaction design optimization.

According to the data described in Table 1, users in the three regions were relatively satisfied with the interaction waiting design. Among the satisfied groups, there were 37 users in Region 1, accounting for 31.4% of the group; there were 40 users in Region 2, accounting for 33.9% of this group; there were 41 users in Region 3, accounting for 34.7% of this group. Among the general group, there were 8 users in Region 1, accounting for 38.1% of the group; there were 6 users in Region 2, accounting for 28.6% of this group; there were 7 users in Region 3, accounting for 33.3% of this group. Among the dissatisfied groups, there were 5 users in Region 1, accounting for 45.5% of the group; there were 4 users in Region 2, accounting for 36.4% of this group; there were 2 users in Region 3, accounting for 18.2% of this group. On the whole, the satisfied group accounted for 78.7% of the total number of respondents; the general group accounted for 14% of the total number of respondents; the dissatisfied group accounted for 7.3% of the total number of respondents. Satisfied users thought that under this design, the interactive waiting time of the product would be shorter, and there would be corresponding expected time prompt in the waiting process, which would not appear dull. Unsatisfied users thought that although their needs have been met after the product interaction waiting for the experience design, the interface design of the product still has defects, especially the frequent pop-up windows during loading. Then, it can analyze the proportion of user waiting time and the effect of information feedback under the mobile Internet product Interaction design. The paper investigated three products, including the traditional product Interaction design before optimization and the interactive waiting design under the mobile Internet after optimization. The three products are mobile phones, sports watches and heart rate collectors, as shown in Fig. 5.

**Figure 6:**
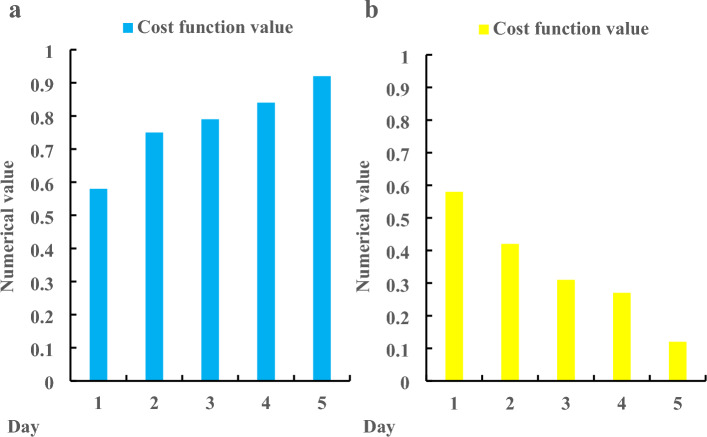
Change of cost function value before and after product interaction design optimization. (**a**) Before product interaction design optimization. (**b**) After product interaction design optimization.

Figure 5a shows the product interaction design before optimization, and Fig. 5b shows the product interaction design after optimization. According to Fig. 5a, before the optimization of product interaction design, the waiting time of Product 1 accounted for 0.24, and the information feedback effect was 0.48; the proportion of waiting time of Product 2 was 0.18, and the effect of information feedback was 0.55; the proportion of waiting time of Product 3 was 0.26, and the effect of information feedback was 0.53. According to Fig. 5b, after the optimization of product interaction design, the waiting time of Product 1 was 0.1, and the information feedback effect was 0.79; the proportion of waiting time of Product 2 was 0.08, and the effect of information feedback was 0.76; the proportion of waiting time of Product 3 was 0.04, and the effect of information feedback was 0.81. On the whole, the proportion of waiting time before product interaction optimization was 0.23, and the effect of information feedback was 0.52; the proportion of waiting time after product interaction optimization was 0.07, and the effect of information feedback was 0.79. Through comparison, the waiting time after product interaction optimization was 0.16 lower than that before optimization, and the information feedback effect was 0.27 higher than that before optimization. The decrease of waiting time indicates that the hardware performance of the product has been greatly improved after optimization, and the interface and product information are also more perfect. This paper then used machine learning to analyze the change of the value of the cost function before and after the optimization of the product interaction design, and investigated the change in 5 days. The specific change is shown in Fig. 6.

**Figure 7:**
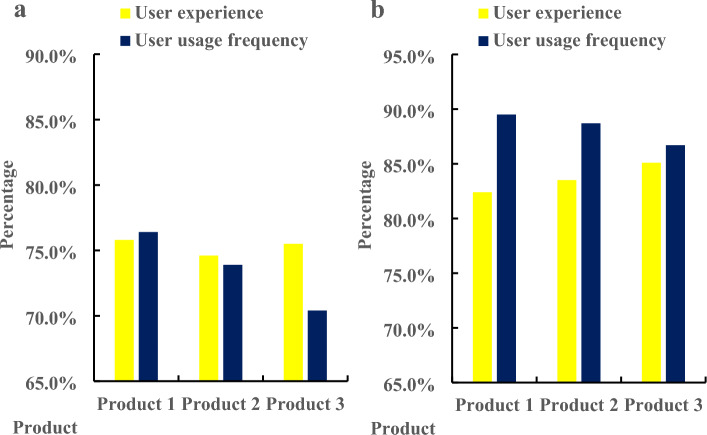
User experience and user use frequency of mobile internet products before and after interactive waiting experience design. (**a**) Before product interaction design optimization. (**b**) After product interaction design optimization.

Figure 6a shows the product interaction design before optimization, and Fig. 6b shows the product interaction design after optimization. According to Fig. 6a, before the optimization of product interaction design, the initial value of the cost function value was 0.58, which increased to 0.92 on the fifth day, and the whole process increased by 0.34, with an average of 0.78. It can be seen from Fig. 6b that after the optimization of product interaction design, the initial value of the cost function value was 0.58, which decreased to 0.12 on the fifth day, and the whole process decreased by 0.46, with an average value of 0.34. It can be seen from the comparison that the cost function value after the optimization of product interaction design was 0.44 lower than that before the optimization. In the optimized product design, the user’s sense of experience would become stronger, and the user’s stickiness would also be greater. Finally, the user experience and user use frequency before and after the interactive waiting experience design of mobile Internet products were analyzed. Three products were investigated, and the specific results were shown in Fig. 7.

Figure 7a shows the changes before the design of product interaction experience, and Fig. 7b shows the changes after the design of product interaction experience. According to Fig. 7a, before the optimization of product interaction design, the user experience of Product 1 was 75.8%, and the user use frequency was 76.4%; the user experience of Product 2 was 74.6%, and the user use frequency is 73.9%; the user experience of Product 3 was 75.5%, and the user frequency was 70.4%. According to Fig. 7b, after the optimization of product interaction design, the user experience of Product 1 was 82.4%, and the user use frequency was 89.5%; the user experience of Product 2 was 83.5%, and the user use frequency was 88.7%; the user experience of Product 3 was 85.1%, and the user frequency was 86.7%. On the whole, before the optimization of product interaction design, the user experience of the product was 75.3%, and the user use frequency was 73.6%. After the product interaction optimization, the user experience of the product was 83.7%, and the user use frequency was 88.3%. Through comparison, the user experience after the product interaction optimization design was 8.4% higher than that before the product interaction optimization, and the user use frequency was 14.7% higher than that before the product interaction optimization.

Through the comparative analysis of the experiment, it can be seen that after the interactive waiting experience optimization of mobile Internet products, the user’s sense of experience is enhanced. Its use frequency is also more frequent, and the user’s demand is stronger. Moreover, after the optimization of product interaction, the information feedback effect and hardware skill problems have been solved and improved. To a certain extent, the user’s tolerance for the product has also been improved, and the user who has waited for a long time can be compensated in time, thus reducing the user’s complaint rate.

## Conclusions

Due to the objective factors when mobile applications interact with users, interactive waiting is inevitable. This article introduced the user interaction feature of waiting for feedback when using mobile applications. During the interactive waiting period, if the user can actively control the required waiting process and successfully complete the specified work task, the user can move on, control and continue at any time, and can feel the right to terminate and callback. The user would have a better feeling when waiting for the experience. Only by deeply understanding the user characteristics of mobile Internet products can they develop mobile Internet products that meet the needs of modern users. When designing the user experience environment, people can ensure consistent and personalized use and provide help and information to users in time.

## Data Availability

Data is available upon reasonable request, if someone wants to request the data from this study please contact Yi Yu (2014010010@hzvtc.edu.cn).
